# American Academy of Dental Science

**Published:** 1896-02

**Authors:** 

**Affiliations:** American Academy of Dental Science


### Reports of Society Meetings.

### AMERICAN ACADEMY OF DENTAL SCIENCE.

The regular monthly meeting of the American Academy of Dental Science was held at Young's Hotel, Boston, October 2, 1895, at six o'clock, President Smith in the chair.

*President Smith.*—Gentlemen,—The essayist of this evening certainly needs no introduction to the Academy. I can only say that it gives me great pleasure to present to you Dr. George F. Grant, who will read a paper on "Hypnotism: Its Value to the Dental Specialist."

(For Dr. Grant's paper, see page 83.)

DISCUSSION.

*President Smith.*—Will Professor Fillebrown kindly open the discussion.

*Dr. Fillebrown.*—I thank the president for the opinion that I may know something about this subject and be ready to speak about it. I have pronounced opinions upon it, but there doesn't seem to be much latitude left for me to express them, as, according to the paper, dentists are debarred from using suggestions at all.

The paper is an excellent *résumé* of what has been said upon the subject. I hoped that the essayist to-night was to give us something practical,—something from his own experience. However, I am very glad that the subject has excited interest enough to bring out a paper from any one aside from myself. I think that a very distinct gain.

I believe that the modern dentist, and particularly a member of this Academy, is something more than a mere tooth-puller or a tooth-plugger; that in his daily work he considers other conditions of the system and the relations of the teeth to them.

I believe that a dentist is bound to know enough to use an anæsthetic; I think he is bound to know when and how to use a narcotic; I think he is bound to know enough to be ready to meet the emergencies that may occur during his dental operations in whatever form they may come. Now, I say that a dentist who does not know how to use every kind of a sedative, or who is not able to comprehend and get a practical knowledge of a matter that is as well understood as mental suggestion is to-day, is not fully equipped for his profession. We are bound by our relations to ourselves, by our relations to our patients, by our professional relations to the community, to be that much of medical men in order to do our duty to our patients. Now, as medical men, I say we are bound to know something of mental suggestion and of its power, and I, for one, offer no excuse for presuming to know what are the accidents that may occur and what I have to guard against. I say that I believe it is our duty to do that, and from that basis I proceed to say something of what I know about hypnotism.

I do not pretend to be an authority for others in this matter, but I do believe that three years constant study of suggestion, using it daily in my practice and reading every author that I could get hold of, is sufficient to make me an authority for myself. I believe I have a fair understanding of the subject. In the works of such authors as Bernheim, Moll, Luys, and Charcot we find the same opinions are repeated; all quote the same facts; they all agree in their description of the same conditions; they all acknowledge the same dangers; they all recommend the same remedies for the same dangers; and when writers agree so fully, their opinions on any matter should not be difficult to understand. In fact, I believe that to-day there is not a therapeutic agent whose actions, limitations, and possibilities are better defined than that of mental suggestion, and there is not a single remedial agent that a man can use any more intelligently than this. If you prohibit the use of suggestion by a dental surgeon, what right has he to use opium, chloroform, or nitrous oxide gas? Here sits a man that, willing or unwilling, is one of the most successful practitioners of the art to-day, and a suggestion that he once made did me more good than anything that I got out of any book. Though I have been using suggestions for several years in my practice, I do not think that for a year and a half I have, in the sense spoken of here to-night, hypnotized a patient, and yet every one that was at all willing received the benefit of suggestion.

I was speaking to Dr. Andrews a moment ago of a patient that was in my chair on Monday. Three years ago I relieved her from a terror of dental operations, which was so powerful that she found it impossible to approach a dentist's office. She was in my office last Monday to have a tooth filled, and it had been so long since her last visit that she was again quite nervous. I felt her pulse and found her heart beating hard. After suggestion for a little time, I prepared and filled the cavity, with no pain and with very little disturbance.

I will mention another case. A young girl, twelve years old, very nervous indeed; she had been under treatment for nervous disturbance; she came to my hand at the close of the day, after trying for two hours to submit to the extraction of a tooth with gas; and could not be induced to even breathe the gas; and was then nearly in hysterics and her mother sadly disappointed. By my use of suggestion in two minutes she ceased sobbing and sat down in my operating-chair, and in three minutes more she voluntarily and quietly opened her mouth and allowed me to extract a first molar without flinching.

You may judge of her mother's astonishment and delight.

The action of the mind in making a suggestion has been made very plain by Dr. W. H. Myers, of London, an active member of the European Society for Psychical Research. In the transactions of that society there are, perhaps, one hundred pages in all devoted to his discussion of the matter. He showed very clearly that the mind has two layers as it were,—a liminal or conscious layer and a subliminal or unconscious layer. One is the mind that we know about in which all acts of volition originate; the other, where all habits lie that we do not directly control. He contends that it is through the communication of one subconscious mind with the other that thought transferrence takes place. My own experience seems to give evidence of the truth of this phenomenon. If I am standing by my patient and want to produce a feeling of repose in my patient, I first want a reposeful feeling myself, and that voluntary action of mine will set my subliminal consciousness at work, and it seems to me that gives origin to the same thought and a similar relation between the liminal and subliminal consciousness of the patient, and in one, two, or maybe five minutes that same reposeful feeling possesses the patient. That is hypnosis, but not to a degree sufficient to effect the consciousness. I don't want to take that into consideration at all; it is the bugbear of being put beyond the limits of their own willpower that so many people object to, but I do not need or want to effect the consciousness or will in the least. All I want to do is to remove the fear of the operation and alleviate the pain of cutting the tooth. Medical practitioners will tell you that many diseases are largely exaggerated by fear, and if in these cases you can remove fear, you can generally remove the pain. In nine cases out of ten hypnotic suggestion answers this purpose.

I have now given you specimens of my experiences, and could give you a volume if there were time. I want to hear what Dr. Stevens can tell us of his experience.

*Dr. Stevens.*—There is this idea that I would suggest: if all dental practitioners were honest men, it might be safe to accept hypnotism for our purposes. If all practitioners were like Dr. Fillebrown, and did not care about carrying the suggestion beyond the stage in which dental operations could be performed without discomfort to the patient, it would be all right. But they are not all like that, and I am afraid it would be bad for the reputation of dentistry to introduce hypnotism into our practice, and my advice is, don't.

*Dr. Fillebrown.*—That does not quite answer my question. I know that Dr. Stevens does exert an influence over his patients more beneficial than most operators, and can perform dental operations for many that are unable or unwilling to undergo the operation, under ordinary conditions. How practical does he make it?

We need not call it hypnotism, call it anything; but will the doctor tell us what he does, and how he succeeds with it.

*Dr. Stevens.*—If I have a patient who is extremely nervous, I try, through my will to control that nervousness; I try to calm their nerves and quiet them; to get them into that condition in which they will allow me to operate without interference, and I have often succeeded.

*President Smith.*—Not in all cases?

*Dr. Stevens.*—No, not always; but I have many times succeeded in that way when I am confident I could have done nothing with other means at my command. When the thing can be done, within limits, it is all right. Of course if a patient is susceptible to hypnotism, in most cases it can be carried to almost any extent. I do not know what Dr. Fillebrown referred to when he spoke, unless it was the use of mental suggestions rather than verbal.

Many times I have had children who have been extremely nervous, and it was almost impossible to do anything with them before my suggesting to them to "let go of themselves," not to resist, to relax the muscles. That is half the battle; you must try to get them to give themselves up entirely, and many of my patients have had operations performed without exhibiting any nervousness at all, who at first would not allow me to put an instrument in their mouths without their screaming.

*Dr. Fillebrown.*—A good many years ago, when I was young and enthusiastic, I happened to be in Boston, and I heard that Dr. Wm. H. Atkinson was to give a clinic at the office of Dr. Dickerman, in Taunton. I wanted so much to see him that I took the trouble to go down there. The patient on whom he was about to clinic was a young boy, and his teeth were desperately sensitive; the cavity was in an inferior right second molar, and it was with great difficulty that any one could get the boy to allow an examination of it. The doctor had great ability to operate without hurting patients. After talking quietly to the lad for some minutes he took his instruments and went to work and cut that cavity all out, and the boy never whimpered. Dr. Ambrose Lawrence was there, and in his brusque way of speaking asked Dr. Atkinson, "How do you do it?" The doctor, with a quiet smile, replied, "Oh! the angels help me." "Yes," says Dr. Lawrence, "we all know that, but *how* do *you* do it?"

Now, Dr. Stevens, how do you do it? You told us that you try to calm their nerves and that you succeeded; and you went on and said that you did it through your will power, but what we want to know is, *How* do you do it? Can it be formulated? Can the rest of us do it?

*Dr. Stevens.*—I cannot tell you.

*Dr. Fillebrown.*—I believe I did tell you when I described the action of the subliminal consciousness. I believe that is the explanation of Dr. Atkinson's success as an operator, not only as a dentist, but as a surgeon. I have heard that when he was about to perform an operation in oral surgery, the first thing he would do would be to get the patient a cup of tea, sympathize with him, tell him that one good thing about an operation of that kind was that it didn't pain much, and in a short time bur out the necrosed bone, and the patient scarcely flinched at all and thought the operation a relief. Dr. Atkinson had a subliminal consciousness of wonderful power, and could control almost everybody around him. Many of us could not expect to meet with the success that he had, but if we study the subject carefully, and acquire the knowledge that is now available, I think we will have little trouble in successfully using it.

Not long ago a friend of mine had a patient, a young boy, that would faint dead away the moment his teeth were touched. Knowing something about what I had done in this line, he asked me if I could do anything with a case of that kind, and I told him to send the boy to me, and I would try. He was glad to get rid of him, and the boy came to me. The first time he felt quite faint. I did not want to hurry matters, so I made another appointment; the second visit he got along very well, the third time a little better, and the fourth time there was no trouble whatever, and I was able during the four sittings to do all the work necessary on his teeth.

Dishonest dentists are very few and far between,—certainly among the class who will be interested in this discussion. I do not believe there is any reason at all why we should not interest ourselves in the matter of hypnotic suggestion, and use it for the benefit of our patients,—in fact, I believe we have no right to refuse to investigate any appliance or method which will make it easier for our patients to undergo dental operations.

*Dr. Stevens.*—I will say just one word in regard to what Dr. Fillebrown has brought up,—that is, in regard to the question of having patients become unconscious at all. It seems to me there is a vast difference between giving an anæsthetic or a narcotic and producing hypnotism. An anæsthetic can now be given without a person's knowledge or consent; hypnotism can be produced without their consent and sometimes without their knowledge. A person who has once been hypnotized can readily be brought under hypnotic influence again, and even at a distance.

*Dr. Fillebrown.*—Such cases are very rare.

*Dr. Grant.*—That is one point that I omitted, because I did not write the paper with any idea of influencing those who understood it not to use it, but merely to examine into its practical value to us. What Dr. Stevens has said is substantiated by every writer, friend or foe, on hypnotic suggestion. Of course, at the present day hypnotic suggestion is generally regarded as a perfectly possible thing, and almost every writer takes especial pains to speak of its dangers and to warn operators against them, even of careless suggestion, which is of course a lesser evil. And they all say that persons who can be hypnotized become more susceptible, and that they will obey even careless suggestions. Dr. Osgood, who has said more about it than any one around here, takes particular pains to say that he sometimes insures patients against the danger that he recognized they are liable to by saying to them in the hypnotic state, "No one but I can ever hypnotize you again." It does not seem necessary to take such precaution if he did not recognize the possibility of danger to the patient. Dr. Fillebrown is the only one who has written on the subject who does not admit that it is a dangerous influence to deal with; all the people who do it professionally agree upon that point.

*President Smith.*—I would like to ask some of you gentlemen who seem to understand this subject, if it is a conceded fact that if a person who produces this hypnotism on a patient should say to that person while in the hypnotic state, "You can never be hypnotized by any one but me," that the patient would not be susceptible to the influence of any other hypnotist?

*Dr. Fillebrown.*—That is true.

*Dr. Grant.*—Yes, it is a fact.

*Dr. Fillebrown.*—All our discussion about dangers will not hinder dishonest people from practising hypnotism. Again I say, is that any reason why we should refuse to give our patients the benefit of it in this mild form? These cases that have been mentioned here to-night are the extreme instances,—they are the one of a thousand. Charcot said that there was not more than one out of a thousand subjects that could be hypnotized without their knowledge or consent.

I will quote Professor William James; none will question his knowledge or familiarity with this subject. He told me himself that he thought it not dangerous to the patient, providing there were honest operators, this provision being necessary in the use of any remedy. I talked with him a little in regard to my use of it in professional life, and my teaching it to others, and asked if he would advise it, and he said, "Yes, by all means, do it." So you see there is a difference of opinion as regards the danger connected with it. I do know there is no unavoidable danger in it, and any one that refuses to use it is not giving his patients all the ease that is in his power to give them.

*Dr. Stevens.*—Of course, I agree with Dr. Fillebrown in this matter of hypnotic suggestion to a certain extent, but I do not think that Dr. Fillebrown has taken into consideration the full force of this matter,—I mean in those cases where the patient becomes unconscious, and I could relate two or three cases in point which will show the degree to which people are sometimes affected.

Some years ago, when I was a student, a young lady came into the office to have a tooth extracted. The lady was a stranger to me and wished to take gas. I took out the tooth and she came out of the gas apparently all right, and after she had rinsed the blood from her mouth, she lay back in her chair in a sleepy, semi-conscious condition. My preceptor was there at the time, and he felt of her pulse and said there was nothing to cause alarm, but she remained in this state for some little time, and when she finally came out of it, and got ready to go, my preceptor thought I had better go home with her. She lived out of town, but I got her home all right. I heard nothing from her until, two or three years afterwards, when I was in practice by myself, she came into my office with a friend. I performed an operation for the friend and she sat in the reception-room reading. Just before completing the operation I had occasion to step into the reception-room, and after a little conversation with her,—at which time she appeared all right,—I returned to my patient and finished what I was doing. Returning to the reception-room, we found her lying back in her chair in a state similiar to that into which she relapsed on her first visit to me. I asked her friend if she knew whether "the lady had been hypnotized at any time," and was informed that she had been. I did not know how to restore her to consciousness. She sang us some "Trilby" songs and seemed to be quite comfortable, and unconcerned as to where she was. She missed the train they had intended to take, and as the time passed on I suggested in a confident manner that she would be ready to take the next train (fervently hoping that she would), and sure enough she was all right in time. Now, what caused that hypnotic state? I knew nothing about hypnotism at the time, but I believe now my suggestion threw it off.

I have another case in mind. A lady, Mrs. B., an entire stranger to me, came into my office to have her teeth examined, and I found that one of the under teeth was beyond all help and needed to be extracted. When I finished the examination I noticed that she appeared to be sort of indifferent to what was going on, and she laid back in the chair with a rather absent-minded expression. I told her, "There is one tooth there which needs to be taken out." Without manifesting much interest in it, she said, "Take it out;" and I did so, without any pain to the patient. That was also before I knew anything about hypnotism, but I thought of this incident afterwards when she came again in the office, and the opportunity was given me to try an experiment. She had a bottle of tooth-powder, and when she put it down, she said to me, "Now, don't you let me forget that tooth-powder." I replied, earnestly, "You cannot pass without picking it up." A few days afterwards she came in and said, "Doctor, I wish you would fix matters up between me and that bottle of tooth-powder. I can't get by it without picking it up." I told her she would not be troubled any more, and she was not. I might add that she was one of that kind who always scouted the idea of being hypnotized, and thought any one was dreadfully weak-minded to allow themselves to be so influenced.

*Dr. Grant.*—I would like to ask Dr. Stevens if he took any pains to acquire the art of suggestion?

*Dr. Stevens.*—Never; except that I have read considerable about it of late years. I do not claim that that was any power of mine.

*Dr. Grant.*—The point I wanted to make was that it is claimed by some that people must have a special training—not only a medical training, but a special training in the science of hypnotism—to acquire the power of suggestion, but it is my belief that it exists in some people to an extraordinary degree. I had a talk with a man who has done quite a little in it, and I asked him that questien especially,—whether he considered it an art or a gift. He said that he could not say; he had known men to acquire a slight degree of training, while others seemed to be able to exert a great deal of influence without giving the subject much thought or study.

*Dr. Fillebrown.*—The very fact of its being possible for one person to hypnotize another without their knowledge is an additional reason for every one understanding it. If Dr. Stevens had understood his own case, all that trouble would have been averted.

A lady was in my chair last Saturday, and we were speaking of hypnotism, and she told me of a young lady who, when she came into her presence, would go into an hypnotic trance. She did not know at first that she caused it; but she found it out finally, and when the young lady again became hypnotized, she remarked that she could take her out of it, and she went up to her and made some reverse passes and she immediately recovered her usual conditions. Now, this power exists, and it is a great deal better that we understand it. Such a case would not be found often enough to become a factor to be taken into consideration, but that it is sometimes possible is one of the strongest reasons why we should understand such conditions; they are forced upon us without our knowledge or consent.

I wish to say one word in regard to the appreciation of it by the profession at large and the increase of interest in this matter. My first account of my experiences in the use of it were published in the *Dental Review.* I had that article with me at the meeting of the American Association in August, 1893, and it was suggested that it be read as a volunteer paper, and the feeling in regard to it then in a committee was that the subject was not relevant, and they were quite unwilling that it should come before the Association. Mark the change that has taken place since. The next year a paper on the same subject was made one of the four that was read before the general meeting of the World's Dental Congress. At the meeting of the American Dental Association in 1894 the subject was not formally considered, but I was solicited to give a clinical talk upon the subject.

Thirty or more dentists gathered and listened, and among them were members of the committee who refused to consider it two years before. This summer I was at Asbury Park attending the meeting of the Association. Nothing was said about suggestion in the meeting, but the same request was made for a clinic, with similar results. The appreciation of it and the practice of it is steadily extending knowledge of its use and of the benefits derived from its interviews from every quarter of the globe. We must not be behind in the adoption of an adjunct which is of undoubted merit.

*President Smith.*—If the members do not care to speak further on this subject, I will ask Dr. Grant to close the discussion.

*Dr. Grant.*—The paper excited some of the discussion which I hoped it would bring up, but I am still of the opinion that after all there is something more in the mere theory of repose and confidence in the operator than we are apt to give credit for, and that this is sometimes mistaken for hypnotic suggestion, and I have not yet heard anything that changes my views of it. When we are masters of ourselves, we are, in a large measure, masters of our patients. I have been in practice now for twenty-five years, and I have this to say, that though I do not profess any power more than is possessed by any other man, I have yet to know of a patient leaving my office because I was unable to perform any operation that I undertook. It may have been good fortune to have had patients who could be handled without the aid of hypnotic suggestion; at any rate, all things considered, I think I have got along very well without attempting to use something whose effect is still uncertain and unknown.

William H. Potter, D.M.D.,
*Editor American Academy of Dental Science.*

